# Novel GATA6 Mutations in Patients with Pancreatic Agenesis and Congenital Heart Malformations

**DOI:** 10.1371/journal.pone.0118449

**Published:** 2015-02-23

**Authors:** Christina S. Chao, Kristen D. McKnight, Kenneth L. Cox, Anne L. Chang, Seung K. Kim, Brian J. Feldman

**Affiliations:** 1 Department of Pediatrics, Stanford University School of Medicine, Stanford, CA, United States of America; 2 Department of Developmental Biology, Stanford University School of Medicine, Stanford, CA, United States of America; 3 Department of Dermatology, Stanford University School of Medicine, Stanford, CA, United States of America; 4 Howard Hughes Medical Institute, Stanford University School of Medicine, Stanford, CA, United States of America; 5 Cardiovascular Institute, Stanford University School of Medicine, Stanford, CA, United States of America; 6 Program in Regenerative Medicine, Stanford University School of Medicine, Stanford, CA, United States of America; NIDCR/NIH, UNITED STATES

## Abstract

Patients with pancreatic agenesis are born without a pancreas, causing permanent neonatal diabetes and pancreatic enzyme insufficiency. These patients require insulin and enzyme replacement therapy to survive, grow, and maintain normal blood glucose levels. Pancreatic agenesis is an uncommon condition but high-throughput sequencing methods provide a rare opportunity to identify critical genes that are necessary for human pancreas development. Here we present the clinical history, evaluation, and the genetic and molecular analysis from two patients with pancreatic agenesis. Both patients were born with intrauterine growth restriction, minor heart defects and neonatal diabetes. In both cases, pancreatic agenesis was confirmed by imaging studies. The patients are clinically stable with pancreatic enzymes and insulin therapy. In order identify the etiology for their disease, we performed whole exome sequencing on both patients. For each proband we identified a de novo heterozygous mutation in the GATA6 gene. GATA6 is a homeobox containing transcription factor involved in both early development of the pancreas and heart. In vitro functional analysis of one of the variants revealed that the mutation creates a premature stop codon in the coding sequence resulting in the production of a truncated protein with loss of activity. These results show how genetic mutations in GATA6 may lead to functional inactivity and pancreatic agenesis in humans.

## Introduction

Pancreatic agenesis is a rare disease with individual and compiled case reports totaling approximately 77 cases with the largest contribution from Allen et al. in 2012 [1–4]. However, this number is likely an underestimate of the total incidence, as many cases are likely unrecognized either due to rapid perinatal mortality, or phenotypic variability [4–6]. To survive, patients with complete pancreatic agenesis require early identification and treatment of neonatal diabetes with insulin and pancreatic enzyme replacement therapy shortly after birth. Even with therapy, Baumeister *et al*. originally reported only 31% (4/13) infant survival in the neonatal period, with deaths attributed to bronchopneumonia or complications from other congenital anomalies [1]. Most patients (92%, 38/41 total cases) with pancreatic agenesis are born with intrauterine growth restriction (IUGR), reflecting a requirement for fetal insulin to support in utero growth [2,5,7–22]. In addition, pancreatic agenesis has been associated with malformations of the biliary, cardiac, intestinal, and nervous systems, with gall bladder agenesis as the most common biliary abnormality [1–3,8,13,16,18,23–25]. Congenital heart defects associated with pancreatic agenesis include atrial septal defect, ventricular septal defect, tetralogy of fallot, pulmonary stenosis, transposition of great vessels, tricuspid atresia, and double outlet right ventricle (S1 Table) [1,2,8]. Elucidating novel mutations that cause pancreatic agenesis are needed to further our understanding of the relationship between genotypes and these associated anomalies, and to provide insight into the cause of the variable clinical courses between patients.

Mutations affecting transcription factors critical for pancreas development have been implicated as the basis of human pancreas agenesis. This includes mutations in the coding region of pancreatic duodenal homeobox 1 (Pdx1; also known as IPF1), and mutations in the locus encoding Pancreatic transcription factor 1A (PTF1A) [14,15,25–27]. In mice, two GATA Binding proteins, Gata4 and Gata6, have been shown to regulate pancreatic and cardiac development [31–34]. Shaw-Smith et al (2014) showed that a heterozygous mutation of *GATA4* was linked to a case of complete pancreatic agenesis [28]. Recently, Allen et al. used next generation exome sequencing and discovered that 15/27 (56%) in one series of patients with pancreatic agenesis had de novo heterozygous mutations in the *GATA6* gene [3]. In these patients with GATA6 haploinsufficiency, 92% (23/25) also had congenital heart defects (S1 Table) [3,4,6,9,16,21]. Thus, Pdx1, Ptf1a, Gata4 and Gata6 are crucial early regulators of pancreas development in mice, and the discovery of loss-of-function mutations affecting these genes in patients with pancreatic agenesis indicates that the developmental functions of these transcription factors may be conserved in humans [29–32].

In humans, heterozygous loss-of-function GATA6 mutations have been linked to pancreatic agenesis, but in mice, Gata6 heterozygous null mutant mice appear phenotypically normal and do not have abnormal pancreas development. In mice, prior studies show that Gata6 and the paralogue Gata4 are expressed and crucial for heart and pancreas development [33–36]. Simultaneous conditional inactivation of Cre-recombinase sensitive alleles of Gata6 and Gata4 resulted in mice with pancreatic agenesis [34,37]. Thus, the requirement for GATA6 function may be distinct in human and rodent pancreatic development, emphasizing the need for functional studies of *GATA6* genetics in human pancreas development.

Here we describe the clinical history, evaluation and molecular studies of two patients with pancreatic agenesis. We performed whole exome sequencing and identified two previously unreported variants in the GATA6 gene. We discovered that one patient has a single base pair change that alters the splice site at exon 4 to 5 intron/exon boundary and the other has a seven base pair nucleotide deletion that results in a frame shift and premature stop codon. We demonstrate that the premature stop results in a truncated GATA6 protein, leading to loss-of-function in transcription assays. In both probands, the novel GATA6 variants are *de novo* and heterozygous, similar to pervious GATA6 variants found in pancreatic agenesis, supporting a mechanism of haploinsufficiency in humans that is distinct from rodent models.

## Methods

### Human subject research and ethics

Skin biopsies and/or whole blood were obtained from proband (n = 2), siblings (n = 2) and parents (n = 4) using an approved IRB protocol from Stanford University. Written informed consent was obtained from each participant at the time of skin and/or blood specimen collection. Both probands were male and one proband had one sister and one brother, which totaled to three female and five male participants. All participants were of Eastern European decent.

### Whole exome sequencing

Skin biopsies were obtained from all participants. Fibroblast cell lines were established from patient skin biopsies and grown in sterile culture media (DMEM, 10% FBS, 1x Penicillin/Streptomycin, and 1ug/uL Fungizone). Whole genomic DNA was isolated from fibroblast cells (Gentra Puregene Cell Kit; Qiagen) and whole blood (PAXgene Blood DNA Kit; Qiagen). Exome sequencing was performed using Agilent SureSelect Human All Exon v4–51Mb kit and HiSeq2000 and bioinformatics was done using raw sequence alignment with human genome build 19 using BWA software to analyze for SNPs and In/Dels (Centrillion Biosciences). Ingenuity Variant Analysis was also used to analyze variant calls. Variants were confirmed using direct DNA Sanger Sequencing. Using previously published primers from Allen et al. in exon 2 and exon 4 PCR products of genomic DNA of probands, siblings and parents were sequenced [3]. In order to Sanger Sequence both alleles PCR products of the probands were subcloned into a TOPO-Blunt II vector (Invitrogen) and colonies were picked and sequenced. The exome sequences will be deposited into dbGaP.

### GATA6 site directed mutagenesis

The variant identified in proband 2 was generated *in vitro* using a wild-type *GATA6* expression plasmid (GATA6 myc-DDK tagged ORF clone; Origene), and Stratagene site-directed mutagenesis (Agilent Technologies). The sequence of the forward primer was ccgctgaacgggaccaccaccacc and the reverse primer was ggtggtggtggtcccgttcagcgg. The wild-type human GATA6 myc-DDK tagged ORF clone was used as the template. Sanger sequencing confirmed the deletion of the ACGT bases resulting in a frameshift mutation and a premature stop codon mimicking proband 2 deletion.

### Luciferase reporter assays

Luciferase assays were performed using the insulinoma cell line INS-1 (gift from Dr. Justin Annes, Stanford University School of Medicine). Cells were cultured in RPM1 1640 with 2mM glutamine and 5% fetal calf serum, 1mM sodium pyruvate, 50 uM 2-mercaptoethanol, 10 mM HEPES, 100 U/ml penicillin and 100 ug/ml streptomycin. Transfections were performed with technical triplicates that were averaged and compared across biological replicates of the experiment, which was repeated six times. Plasmids were transfected into cells using Lipofectamine LTX Plus (Life Technologies) according to the manufacturer’s protocol (4uL per sample reaction). Co-transfections included Hnf4α_P2–2200 promoter (Addgene Plasmid 31062, [38]) (0.1ug) and vector only (pCMV-Tag3), ‘GATA6 wildtype’, ‘GATA6 variant’ (0.5ug) or combined GATA6 wild-type (0.5ug) and GATA6 variant (0.5ug) in 24 well plates. ‘Vector only’ is an empty pCMV plasmid.‘GATA6 variant’ refers to GATA6 with the Proband 2 mutation, *GATA6* ^Y323fsX21^. After 48 hours of incubation, Dual Luciferase Assays (Promega) were performed to measure relative luciferase activity. Relative luciferase activity was normalized to renilla (5ng per transfection) and compared to vector only. We compared firefly/renilla luciferase values for GATA6 wild-type, GATA6 variant, and GATA6 wild-type with GATA6 variant to firefly/renilla luciferase values with vector alone, and calculated statistical significance using a two sided t-test.

### Western blot analysis

Transfections of HEK 293T cells were performed using Polyfect (10 ul per reaction) according to manufacturer’s protocol. GATA6 wild-type or GATA6 variant plasmids (1ug) were transfected in 12 well plates. Cells were lysed in modified RIPA buffer. Protein lysates were run on a 10% SDS-PAGE gel, transferred to PVDF membrane, and probed with mouse anti beta actin (Sigma 1:5000) and goat anti GATA6 antibody (Santa Cruz N-19 sc-7245 1:500). GATA6 antibody required pre-clearing, in which the antibody was initially pre-cleared with protein lysates to eliminate non-specific binding.

## Results and Discussion

### Clinical history

**Proband 1.** Proband 1 is a male born at term gestational age with intrauterine growth restriction via cesarean section due to maternal fibroids. Although his delivery was uneventful, at one hour of life he developed severe respiratory distress requiring intubation and transfer to the neonatal intensive care unit. He developed diabetic ketoacidosis and hyperglycemia, which improved with insulin therapy. Other health conditions in proband 1 include mild anemia, an atrial septal defect (ASD) and Patent Ductus Arteriosus (PDA). On initiation of feeding, exocrine pancreatic dysfunction was discovered, and a subsequent CT scan confirmed pancreatic agenesis. His current medications are insulin and pancreatic enzyme replacement. He is reported to have mild developmental delay. Proband 1 has no siblings. Parental history includes impaired glucose tolerance in the pre-diabetic range with fasting blood sugars between 100–120 mg/dL and post prandial blood sugars above 200 mg/dL in the mother. The father of the proband does not have diabetes, known congenital anomalies or autoimmunity. There is a paternal cousin with biliary atresia who required a liver transplant at the age of five years old.

**Proband 2.** Proband 2 is a male who was born at term gestational age with intrauterine growth restriction. At birth neonatal diabetes was discovered, requiring intensive care for several weeks. His condition improved with insulin therapy. At three months of age, he was noted to have a poor growth velocity and was referred to gastroenterology where he was diagnosed with pancreatic exocrine insufficiency. Ultrasounds performed at 6 months and 13 months of age, both failed to identify any pancreatic tissue but visualization was limited due to bowel gas. At 14 months of age an endoscopic retrograde cholangiopancreatography confirmed that the cystic duct, gallbladder and pancreas were all absent. There were short ductal structures, with two very small branches emanating medially that were thought to be tiny main pancreatic ducts. He was found to have mitral valve stenosis with mild regurgitation and a PDA. The PDA was subsequently ligated. His current medications are insulin, pancreatic enzymes and fat-soluble vitamins. He has an older brother and younger sister. There is no family history of diabetes, autoimmune disease or congenital abnormalities.

Proband 1 and 2 have pancreatic agenesis and congenital heart defects similar to other patients with pancreatic agenesis (summarized in S1 Table) [14,15,25,26]. Both had neonatal diabetes at birth and required ligation of a PDA. Consistent with other patients with pancreatic agenesis, proband 2 also has gall bladder agenesis [3,16,19]. Our report provides additional evidence showing patients born with neonatal diabetes as well as congenital heart defects may require imaging for pancreatic agenesis, and pancreatic exocrine sufficiency testing to exclude pancreatic agenesis as a cause of neonatal diabetes.

### Identification of novel GATA6 variants

To determine genetic variants associated with pancreatic agenesis, high-throughput whole exome sequencing was performed on DNA isolated from probands 1 and 2. Sequencing coverage of the exomes resulted in a total yield of 27.17 megareads and 5.43 gigabases for proband 1, and 39.98 megareads and 7.88 gigabases for proband 2. We performed bioinformatics analysis comparing the variant calls from the probands’ exome sequences to the genome reference consortium human build 19 also known as hg19 (GenBank Assembly ID GCA_000001405.1 http://www.ncbi.nlm.nih.gov/assembly/GCF_000001405.13/ on May 26^th^ 2014).

**Fig 1:**
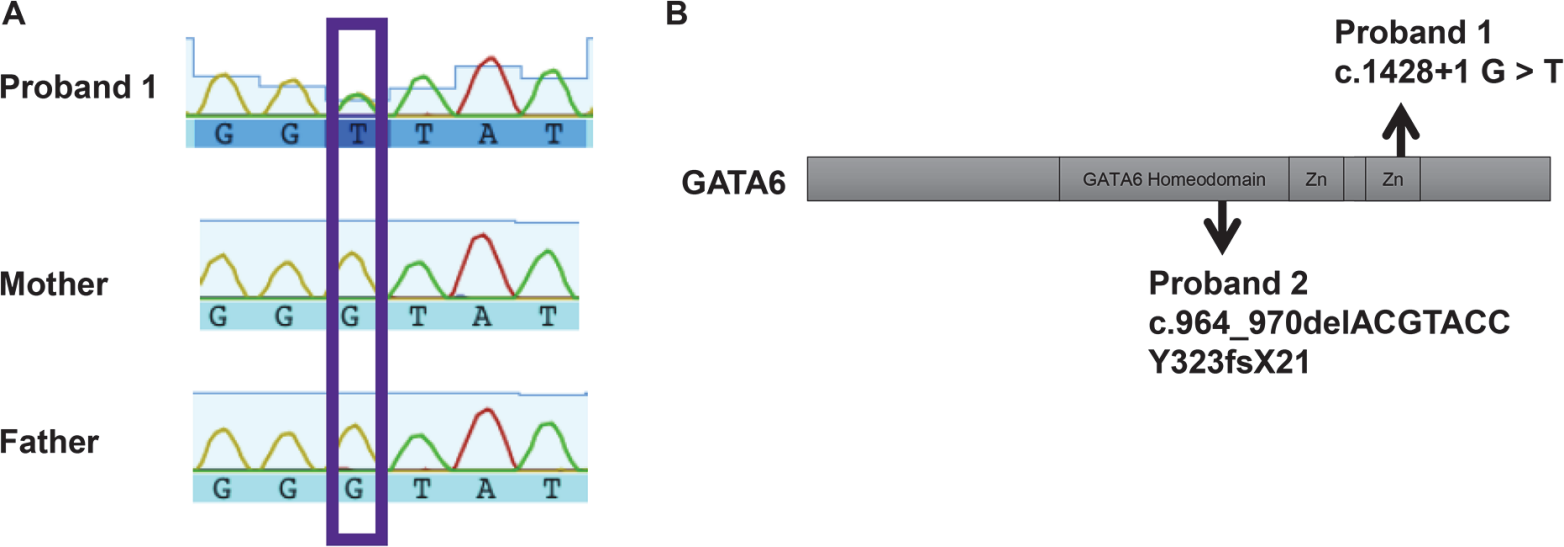
GATA6 variant proband 1. A) DNA sequence of proband and parents showing a single base pair change from G in the parents to a T in the proband at nucleotide position c.1428+1 G>T. B) Diagram of the GATA6 protein containing a GATA family homeodomain and two DNA binding zinc fingers. Arrows indicate locations of GATA6 variants discovered in proband 1 and 2.

Analysis of the exome sequence of proband 1 identified a novel heterozygous G to T transition in GATA6 at position c.1428+1, which was confirmed by Sanger sequencing (Fig. 1A). This nucleotide change in proband 1 is located in the exon 4 splice donor site on GATA6 c.1428+1 G>T. The location of this variant likely abrogates proper splicing, resulting in a run-on transcript with downstream exons out of frame or could possibly result in skipping exon 4, which would truncate part of a zinc-finger DNA binding domain (Fig. 1B). Interestingly, nucleotide changes in GATA6 splice regions have recently been reported in 2 other patients with pancreatic agenesis (De Franco et al (S1 Table, cases 19 and 20) [4]. These types of splice sites disruptions can lead to anomalous alternative splicing and rapid degradation of the transcripts or the resulting truncated protein [39–41].

**Fig 2:**
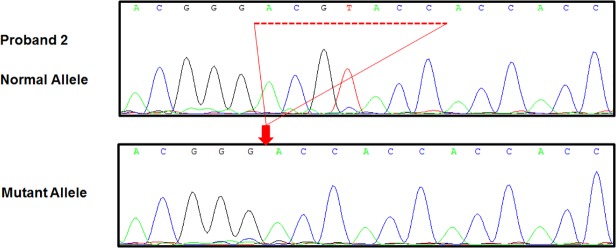
GATA6 variant proband 2. DNA Sanger sequencing of proband 2 demonstrates he has a normal allele (upper) and a mutant allele (lower). The wild-type GATA6 sequence c964_970 (underlined region) is deleted in the mutant allele (arrow).

In proband 2 the exome sequencing identified a de novo heterozygous mutation in GATA6 located at c.964_970delACGTACC. Our sequencing confirmed that proband 2 has a deletion of seven nucleotides (c.964_970delACGTACC) in the GATA6 gene (Fig. 2). Direct sequencing of the PCR amplicons of genomic DNA for proband 2 showed a mixed population, suggesting a heterozygous state. To confirm this, the amplicons were subcloned into a plasmid vector and 8 independent clones were sequenced. We found that four of the clones were the wild-type allele with an intact ACGTACC sequences and the other four were a variant: GATA6 with c.964_970delACGTACC DNA sequences. These results are most consistent with a heterozygous variant allele. In addition, we sequenced both parents and siblings and found that the sequences of the GATA6 gene matched the normal reference sequence in each of these cases (data not shown), indicating that the variant in proband 2 occurred de novo.

**Fig 3:**
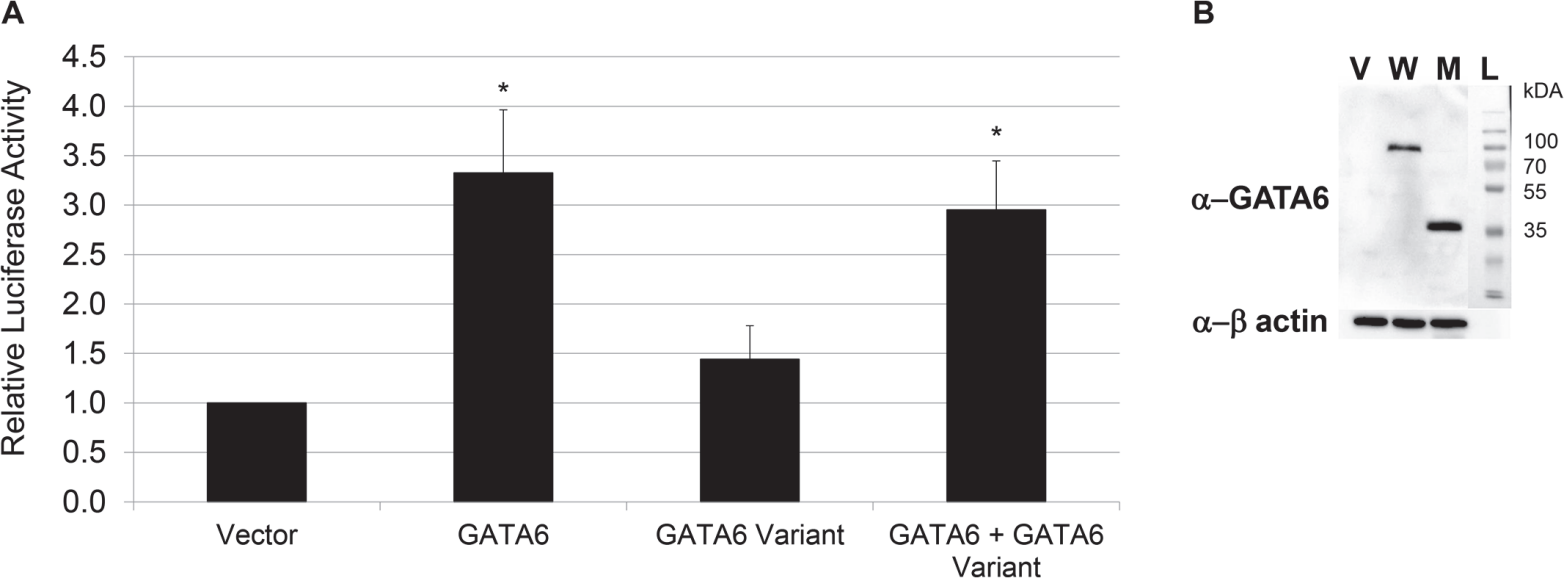
Loss of Hnf4α promoter activation by GATA6 with *GATA6* ^Y323fsX21^. A) Human wild-type GATA6 and/or *GATA6* ^Y323fsX21^ labeled ‘GATA6 variant’ were co-expressed with Hnf4α promoter in INS-1 cells. Hnf4α promoter luciferase activity was measured and levels normalized to vector alone. *P = 0.00001. B) Western blot analysis of human GATA6 protein (W lane) and *GATA6* ^Y323fsX21^ (M lane) expressed in HEK 293T cells. Vector (V lane) only was a negative control and to approximate the size a protein ladder (L lane) was utilized. GATA6 was detected with a GATA6 antibody against the N-terminal of the protein and beta actin was measured as a loading control.

Translation of the c.964_970delACGTACC GATA6 sequence we identified in proband 2 is predicted to produce a frame shift mutation in exon 2 at tyrosine 323 changing it to a threonine (Y323T) and to introduce a premature stop codon 21 amino acids downstream of the frame shift (Y323fsX21) (Fig. 1B). The predicted protein for this variant Y323fsX21 is a truncated GATA6 protein that terminates at amino acid 343, compared to the full-length 595 residue wild-type protein. The deleted C-terminal region of GATA6 contains both zinc finger DNA binding domains and the nuclear localization sequence (Fig. 1B). To test the prediction that Y323fsX21 will produce a truncated protein product, we re-created this variant using site directed mutagenesis in a GATA6 mammalian expression vector. Western blotting revealed that cells transfected with mutant GATA6 generate a truncated protein (Fig. 3B). Thus, the *GATA6* ^Y323fsX21^ allele encodes for a truncated protein.

### Protein produced from *GATA6* ^Y323fsX21^ fails to activate transcription

GATA6 is a zinc finger transcription factor that is important for the development of the hematopoietic, cardiac and gastrointestinal systems. Mouse knockout studies demonstrated that Gata6 directly regulates expression of *hepatocyte nuclear factor 4 alpha* (*HNF4α)*, a crucial regulator of pancreas development and beta-cell function by binding to an enhancer element in *HNF4α* [3,38,42,43]. To test the impact of the *GATA6* ^Y323fsX21^ variant on functional activity, we measured the ability of this variant to transactivate the HNF4α promoter using a reporter construct that contains this GATA6 enhancer element and the endogenous *HNF4*α promoter, preceding a luciferase reporter gene (HNF4α-Luc) [3,38,42]. INS-1 cells were transfected with HNF4α-Luc and wild-type or *GATA6* ^Y323fsX21^ or both and luciferase activity was quantified. We found that wild-type GATA6 transactivates the HNF4α promoter as expected (Fig. 3A). Conversely, *GATA6* ^Y323fsX21^ failed to transactivate HNF4α promoter (Fig. 3A). In addition, co-transfection of *GATA6* ^Y323fsX21^ with wild-type GATA6 did not inhibit HNF4α-Luc activation (Fig. 3A). Thus, in this setting, we did not detect dominant negative activity of the *GATA6* ^Y323fsX21^ variant. In sum, our results confirm that the novel *GATA6* ^Y323fsX21^ variant is likely a loss-of-function mutation that could be caused by either loss of the critical zinc finger domain or from nonsense mediated mRNA decay of the truncated transcript. Further, these results support a mechanism of haploinsufficiency causing disease in humans that is distinct from the rodent GATA6 models [34,37].

Our studies are consistent with previous results that found point mutations in the zinc fingers of GATA6 result in loss of Hnf4α-Luc activation [3]. Here we show the *GATA6* ^Y323fsX21^ variant also results in loss of Hnf4α-Luc activation. There are two previous reports on patients with pancreatic agenesis and *GATA6* mutations where single base pair insertions were predicted to produce a frameshift and premature stop codon resulting in a truncated protein (S1 Table, case 4 and 5) [4,21]. Thus, with our current study, there are three reported patients with pancreatic agenesis who have mutations producing a truncation of GATA6 protein after the Tyrosine 323 residue. Since it is unlikely that all variants in GATA6 will result in functional changes in protein activity, we believe it will be important to assess protein function resulting from nucleotide changes in this gene in order to classify a specific mutation as disease-causing.

## Supporting Information

S1 Table Summary of GATA6 mutations in patients with pancreatic agenesis. (PDF)

## References

[1] Baumeister FA, Engelsberger I, Schulze A (2005) Pancreatic agenesis as cause for neonatal diabetes mellitus. Klin Padiatr 217: 76–81. 15770578 10.1055/s-2004-822657

[2] Chen R, Hussain K, Al-Ali M, Dattani MT, Hindmarsh P, et al (2008) Neonatal and late-onset diabetes mellitus caused by failure of pancreatic development: report of 4 more cases and a review of the literature. Pediatrics 121: e1541–1547. 10.1542/peds.2007-3543 18519458

[3] Lango Allen H, Flanagan SE, Shaw-Smith C, De Franco E, Akerman I, et al (2012) GATA6 haploinsufficiency causes pancreatic agenesis in humans. Nat Genet 44: 20–22. 10.1038/ng.1035 PMC4062962 22158542

[4] De Franco E, Shaw-Smith C, Flanagan SE, Shepherd MH, International NDMC, et al (2013) GATA6 mutations cause a broad phenotypic spectrum of diabetes from pancreatic agenesis to adult-onset diabetes without exocrine insufficiency. Diabetes 62: 993–997. 10.2337/db12-0885 23223019 PMC3581234

[5] Nakao A, Takeda T, Hisaeda Y, Hirota A, Amagata S, et al (2013) Pancreatic agenesis with congenital diaphragmatic hernia and congenital heart disease: a case report. AJP Rep 3: 119–122. 10.1055/s-0033-1353388 24147249 PMC3799714

[6] Suzuki S, Nakao A, Sarhat AR, Furuya A, Matsuo K, et al (2014) A case of pancreatic agenesis and congenital heart defects with a novel GATA6 nonsense mutation: evidence of haploinsufficiency due to nonsense-mediated mRNA decay. Am J Med Genet A 164A: 476–479. 10.1002/ajmg.a.36275 24310933

[7] Ashraf A, Abdullatif H, Hardin W, Moates JM (2005) Unusual case of neonatal diabetes mellitus due to congenital pancreas agenesis. Pediatr Diabetes 6: 239–243. 16390394 10.1111/j.1399-543X.2005.00114.x

[8] Balasubramanian M, Shield JP, Acerini CL, Walker J, Ellard S, et al (2010) Pancreatic hypoplasia presenting with neonatal diabetes mellitus in association with congenital heart defect and developmental delay. Am J Med Genet A 152A: 340–346. 10.1002/ajmg.a.33194 20082465

[9] Bonnefond A, Sand O, Guerin B, Durand E, De Graeve F, et al (2012) GATA6 inactivating mutations are associated with heart defects and, inconsistently, with pancreatic agenesis and diabetes. Diabetologia 55: 2845–2847. 10.1007/s00125-012-2645-7 22806356

[10] Bruce MB, Coutts JP (1982) Complete agenesis of the mid-gut: a case report. Aust N Z J Surg 52: 313–315. 6954935 10.1111/j.1445-2197.1982.tb05408.x

[11] Giannattasio A, Campus R, Muraca M, Lucigrai G, Michelazzi A, et al (2009) Amyand's hernia in a child with permanent neonatal diabetes due to pancreatic agenesis. Pediatr Rep 1: e6 10.4081/pr.2009.e6 21589822 PMC3096026

[12] Gurson CT, Tahsinoglu M, Yakacikli S, Ertugrul T (1970) A case of agenesis of the dorsal pancreas with interventricular septal defect in an infant. Helv Paediatr Acta 25: 522–526. 5493568

[13] Samaee H, Sadeghi-Moghadam P, Arab-Hosseini A, Aramesh MR, Marzban A (2008) Neonatal diabetes mellitus due to pancreatic agenesis. Arch Iran Med 11: 335–336. doi: 08113/AIM.0018 18426328

[14] Schwitzgebel VM, Mamin A, Brun T, Ritz-Laser B, Zaiko M, et al (2003) Agenesis of human pancreas due to decreased half-life of insulin promoter factor 1. J Clin Endocrinol Metab 88: 4398–4406. 12970316 10.1210/jc.2003-030046

[15] Sellick GS, Barker KT, Stolte-Dijkstra I, Fleischmann C, Coleman RJ, et al (2004) Mutations in PTF1A cause pancreatic and cerebellar agenesis. Nat Genet 36: 1301–1305. 15543146 10.1038/ng1475

[16] Stanescu DE, Hughes N, Patel P, De Leon DD (2014) A novel mutation in GATA6 causes pancreatic agenesis. Pediatr Diabetes. 10.1111/pedi.12111 PMC4102676 24433315

[17] Stingl H, Schnedl WJ, Krssak M, Bernroider E, Bischof MG, et al (2002) Reduction of hepatic glycogen synthesis and breakdown in patients with agenesis of the dorsal pancreas. J Clin Endocrinol Metab 87: 4678–4685. 12364458 10.1210/jc.2002-020036

[18] Taha D, Bardise J, Hegab A, Bonnefond A, Marchand M, et al (2008) Neonatal diabetes mellitus because of pancreatic agenesis with dysmorphic features and recurrent bacterial infections. Pediatr Diabetes 9: 240–244. 10.1111/j.1399-5448.2007.00365.x 18547237

[19] Verwest AM, Poelman M, Dinjens WN, Batstra MR, Oostra BA, et al (2000) Absence of a PDX-1 mutation and normal gastroduodenal immunohistology in a child with pancreatic agenesis. Virchows Arch 437: 680–684. 11193482 10.1007/s004280000305

[20] Voldsgaard P, Kryger-Baggesen N, Lisse I (1994) Agenesis of pancreas. Acta Paediatr 83: 791–793. 7949819 10.1111/j.1651-2227.1994.tb13144.x

[21] Eifes S, Chudasama KK, Molnes J, Wagner K, Hoang T, et al (2013) A novel GATA6 mutation in a child with congenital heart malformation and neonatal diabetes. Clinical Case Reports 1: 86–90. 10.1002/ccr3.33 25356219 PMC4184756

[22] Sferruzzi-Perri AN, Vaughan OR, Forhead AJ, Fowden AL (2013) Hormonal and nutritional drivers of intrauterine growth. Curr Opin Clin Nutr Metab Care 16: 298–309. 10.1097/MCO.0b013e32835e3643 23340010

[23] Barbarini DS, Haslinger V, Schmidt K, Patch AM, Muller G, et al (2009) Neonatal diabetes mellitus due to pancreas agenesis: a new case report and review of the literature. Pediatr Diabetes 10: 487–491. 10.1111/j.1399-5448.2009.00523.x 19496968

[24] D'Amato E, Giacopelli F, Giannattasio A, D'Annunzio G, Bocciardi R, et al (2010) Genetic investigation in an Italian child with an unusual association of atrial septal defect, attributable to a new familial GATA4 gene mutation, and neonatal diabetes due to pancreatic agenesis. Diabet Med 27: 1195–1200. 10.1111/j.1464-5491.2010.03046.x 20854389

[25] Thomas IH, Saini NK, Adhikari A, Lee JM, Kasa-Vubu JZ, et al (2009) Neonatal diabetes mellitus with pancreatic agenesis in an infant with homozygous IPF-1 Pro63fsX60 mutation. Pediatr Diabetes 10: 492–496. 10.1111/j.1399-5448.2009.00526.x 19496967 PMC6951802

[26] Stoffers DA, Zinkin NT, Stanojevic V, Clarke WL, Habener JF (1997) Pancreatic agenesis attributable to a single nucleotide deletion in the human IPF1 gene coding sequence. Nat Genet 15: 106–110. 8988180 10.1038/ng0197-106

[27] Weedon MN, Cebola I, Patch AM, Flanagan SE, De Franco E, et al (2014) Recessive mutations in a distal PTF1A enhancer cause isolated pancreatic agenesis. Nat Genet 46: 61–64. 10.1038/ng.2826 24212882 PMC4131753

[28] Shaw-Smith C, De Franco E, Allen HL, Batlle M, Flanagan SE, et al. (2014) GATA4 mutations are a cause of neonatal and childhood-onset diabetes. Diabetes. 10.2337/db14-0061 PMC6850908 24696446

[29] Ahlgren U, Jonsson J, Edlund H (1996) The morphogenesis of the pancreatic mesenchyme is uncoupled from that of the pancreatic epithelium in IPF1/PDX1-deficient mice. Development 122: 1409–1416. 8625829 10.1242/dev.122.5.1409

[30] Jonsson J, Carlsson L, Edlund T, Edlund H (1994) Insulin-promoter-factor 1 is required for pancreas development in mice. Nature 371: 606–609. 7935793 10.1038/371606a0

[31] Offield MF, Jetton TL, Labosky PA, Ray M, Stein RW, et al (1996) PDX-1 is required for pancreatic outgrowth and differentiation of the rostral duodenum. Development 122: 983–995. 8631275 10.1242/dev.122.3.983

[32] Krapp A, Knofler M, Frutiger S, Hughes GJ, Hagenbuchle O, et al (1996) The p48 DNA-binding subunit of transcription factor PTF1 is a new exocrine pancreas-specific basic helix-loop-helix protein. EMBO J 15: 4317–4329. 8861960 PMC452157

[33] Decker K, Goldman DC, Grasch CL, Sussel L (2006) Gata6 is an important regulator of mouse pancreas development. Dev Biol 298: 415–429. 16887115 10.1016/j.ydbio.2006.06.046 PMC2824170

[34] Xuan S, Borok MJ, Decker KJ, Battle MA, Duncan SA, et al (2012) Pancreas-specific deletion of mouse Gata4 and Gata6 causes pancreatic agenesis. J Clin Invest 122: 3516–3528. 10.1172/JCI63352 23006325 PMC3461916

[35] Zhao R, Watt AJ, Battle MA, Li J, Bondow BJ, et al (2008) Loss of both GATA4 and GATA6 blocks cardiac myocyte differentiation and results in acardia in mice. Dev Biol 317: 614–619. 10.1016/j.ydbio.2008.03.013 18400219 PMC2423416

[36] Zhao R, Watt AJ, Li J, Luebke-Wheeler J, Morrisey EE, et al (2005) GATA6 is essential for embryonic development of the liver but dispensable for early heart formation. Mol Cell Biol 25: 2622–2631. 15767668 10.1128/MCB.25.7.2622-2631.2005 PMC1061656

[37] Carrasco M, Delgado I, Soria B, Martin F, Rojas A (2012) GATA4 and GATA6 control mouse pancreas organogenesis. J Clin Invest 122: 3504–3515. 10.1172/JCI63240 23006330 PMC3461915

[38] Thomas H, Jaschkowitz K, Bulman M, Frayling TM, Mitchell SM, et al (2001) A distant upstream promoter of the HNF-4alpha gene connects the transcription factors involved in maturity-onset diabetes of the young. Hum Mol Genet 10: 2089–2097. 11590126 10.1093/hmg/10.19.2089

[39] Lareau LF, Green RE, Bhatnagar RS, Brenner SE (2004) The evolving roles of alternative splicing. Curr Opin Struct Biol 14: 273–282. 15193306 10.1016/j.sbi.2004.05.002

[40] Bingham PM, Chou TB, Mims I, Zachar Z (1988) On/off regulation of gene expression at the level of splicing. Trends Genet 4: 134–138. 2853467 10.1016/0168-9525(88)90136-9

[41] Sorek R, Shamir R, Ast G (2004) How prevalent is functional alternative splicing in the human genome? Trends Genet 20: 68–71. 14746986 10.1016/j.tig.2003.12.004

[42] Morrisey EE, Tang Z, Sigrist K, Lu MM, Jiang F, et al (1998) GATA6 regulates HNF4 and is required for differentiation of visceral endoderm in the mouse embryo. Genes Dev 12: 3579–3590. 9832509 10.1101/gad.12.22.3579 PMC317242

[43] Yamagata K (2014) Roles of HNF1alpha and HNF4alpha in pancreatic beta-cells: lessons from a monogenic form of diabetes (MODY). Vitam Horm 95: 407–423. 10.1016/B978-0-12-800174-5.00016-8 24559927

