# Supplementary material for: Novel GATA6 Mutations in Patients with Pancreatic Agenesis and Congenital Heart Malformations
Source: PLoS One. 2015 Feb 23;10(2):e0118449. doi: 10.1371/journal.pone.0118449 (PMC4338276; doi:10.1371/journal.pone.0118449)
Supplement: S1 Table — (PDF) [file pone.0118449.s001.pdf]

Supplemental Table S1: Summary of GATA6 mutations in patients with pancreatic agenesis

Case	Reference	De novo	GATA6 mutation	Mutant Protein	Heart abnormality	Gall bladder agenesis	Other congenital anomalies	Other endocrine	Cognitive Changes	Relative with same mutation
1	Stanescu 2014	Yes	c.606_609dupGTAC	p.His204ValfsX97	TA	Yes	Left hydronephrosis hydroureter			
2	Allen 2012	Yes	c.701delC	p.Pro234HisfsX60	ASD, VSD					
3	Allen 2012	ND	c.877_880delinsTAC	p.Val293TyrfsX27	TOF		Biliary atresia, inguinal hernia, microcephaly		Learning difficulties	
4	Eifes 2013	Yes	c.968dupA	p.Tyr323fs	ASD, VSD, valvular pulmonary stenosis					
5	DeFranco 2013	Paternal Inherited	c.969 C>CA	p.Y323X	ASD, PDA					Father with adult onset diabetes and VSD
**6**	**Proband 2**	**Yes**	**c.964_970del ACGTACC**	**p.Tyr323fsX21**	**PDA, mitral valve stenosis**	**Yes**				
7	DeFranco 2013	Paternal Inherited	c.1036_1042del	p.T346PfsX44	TOF			Hypothyroid	Mild learning difficulties	Father with adult onset DM
8	DeFranco 2013	Paternal Inherited	c.1136-2A>AG		PDA		Hepatic dysfunction			Father with child-onset diabetes
9	Allen 2012	Yes	c.1108_1121dup	p.Gly375SerfsX22	TOF					
10	Allen 2012	Yes	c1303-10 C>G	p.?	Interrupted aortic arch	Yes				
11	Allen 2012	Yes	c.1354 A>G	p.Thr452Ala	ASD		Colonic perforation		Developmental delay	
12	Allen 2012	Yes	c1366 C>T	p.Arg 456Cys	TOF		Umbilical hernia		Developmental delay	
13	Allen 2012	Yes	c.1367G>A	p.Arg456His	PDA, VSD, hypoplasitc left pulmonary artery				Severe developmental delay	
14	Allen 2012	ND	c.1396A>G		PDA,	Yes	Intestinal malrotation and microcolon	Transient hypothyroidism	Developmental delay and epilepsy	

Case	Reference	De novo	GATA6 mutation	Mutant Protein	Heart abnormality	Gall bladder agenesis	Other congenital anomalies	Other endocrine	Cognitive Changes	Relative with same mutation
15	Allen 2012	ND	c.1399G>A	p.Ala467Thr	ASD, Pulmonary stenosis			Pituitary agenesis	Moderate learning difficulties and seizures	
16	DeFranco 2013	Paternal Inherited	c.1406G>GA	p.G469E	None		Hepatomegaly, diaphragmatic hernia	Hypothyroid	Mild to moderate developmental delay, hemiplegia	Father with adult-onset diabetes
17	Allen 2012	Yes	c.1417A>C	p.Lys473Glin	ASD	Yes				
**18**	**Proband 1**	**Yes**	**c.1428+1 G>T**	**alternative splicing resulting in a run-on transcript with downstream exons out of frame or may result in skipping exon 4**	**ASD, PDA**				**Mild developmental delay**	
19	DeFranco 2013	Yes	c.1429-8 T>TG	predicted to create a cryptic acceptor site at potion -7 which results in inclusion of 7 extra bases causing a frame shit and stop codon after 9 residues	Dextrocardia, Aorto-pulmonary window, AV septal defect		Diaphragmatic hernia		Early cognitive/moor delay	
20	DeFranco 2013	Yes	c.1429-41_1441 del	abolishes the acceptor splicing site and possibility of skipping exon 5	PDA					
21	Allen 2012	Yes	c.1448_1455del	p.Met483ArgfsX11	ASD, mild hypoplasia of right ventricle and tricuspid valve, pulmonary stenosis, PDA, multiple VSDs	Yes				

Case	Reference	De novo	GATA6 mutation	Mutant Protein	Heart abnormality	Gall bladder agenesis	Other congenital anomalies	Other endocrine	Cognitive Changes	Relative with same mutation
22	Nakao 2013/Suzuki	Yes	c.1477C>T	p.Arg493X	VSD, PDA		Diaphragmatic hernia			
23	Allen 2012	Yes	c.1448_1455del	p.Met483ArgfsX11	ASD, mild hypoplasia of right ventricle and tricuspid valve, pulmonary stenosis, PDA, multiple ventricular septal defects	Yes				
24	Allen 2012	Yes	c.1498_1501del	p.Lys500GinfsX14	DOLV, VSD, hypoplastic pulmonary artery, valvular pulmonary stenosis, PFO, PDA					
25	Bonnefond 2012	Yes	c.1504_1505del	p.Lys502spfsX5	VSD	Yes	Bicornuate uterus			Sister with gall bladder agenesis, pancreatic hypoplasia but diabetes
26	Allen 2012	Yes	c.1516+4A>G		None		Left Diaphragmatic hernia			
27	Allen 2012	Yes	c.1516+1 G>C	p.?	ASD, PFO			Transient hypothyroidism		

Abbreviations: Truncus arteriosis (TA), atrial septal defect (ASD), ventricular septal defect (VSD), tetralogy of fallot (TOF), patent ductus arteriosis (PDA), patent foramen ovale (PFO), double outlet left ventricle (DOLV), and atrial-ventricular (AV).
